# Prevention and Intervention with Young People as a Critical Public Health Strategy to Curtail the Opioid Epidemic: A Call to Action

**Published:** 2023-04-27

**Authors:** Carla Kmett Danielson, Jenna McCauley, Jesse Hinkley, Austin Hahn, Angela Moreland, Cristina López, Morgan Goodyear, Zack Adams, Mike McCart

**Affiliations:** 1 Department of Psychiatry & Behavioral Sciences, Medical University of South Carolina, United States; 2 Department of Psychiatry, University of Colorado School of Medicine, United States; 3 Department of Psychiatry & Behavioral Sciences, Indiana University School of Medicine, United States; 4 Oregon Social Learning Center, United States

**Keywords:** Opioid overdose, Opioid use disorder, Public health

## Abstract

Opioid use continues to represent a significant public health problem in the United States, as well as globally. The opioid epidemic has motivated advances in the effective treatment of opioid use disorder (OUD), with a particular focus on medications for OUD (MOUD), including methadone, buprenorphine, and naltrexone. Although these medications are remarkably effective, MOUD expansion initiatives alone have not been sufficient to combat the opioid epidemic. Further, critical questions remain regarding the effectiveness of these medications for individuals who initiate opioid use under age 16. Key strategies to combat the opioid epidemic, including MOUD and naloxone distribution, target intervention for individuals who have already developed an OUD. Like every other health problem, shifting attention earlier in the etiological process can lend itself to a more cost-effective approach by preventing the onset of behaviors that contribute to subsequent increases in morbidity and mortality. Therefore, we argue that targeted interventions for adolescents with substance use problems, including for non-opioid drugs (i.e., cannabis, alcohol), is critical to prevent the onset of OUD and turn the tide of the opioid overdose epidemic. In line with this call to action to move toward earlier intervention as a public health strategy, we propose several concrete recommendations. These include use of universal screening and prevention strategies for teens, an enhanced focus on addressing mental health (i.e., depression, trauma-related anxiety) and ecological (i.e., low caregiver monitoring, affiliating with substance using peers) precursors of substance use initiation in adolescents, a significant restructuring of resource allocation to more effectively and equitably address youth substance use and mental health problems, and continuous efforts dedicated to the de-stigmatization of the disease of substance use disorders.

## Introduction

Opioid overdose and opioid use disorder (OUD) rates are of grave public health concern. Opioid use remains at crisis levels across the globe, with OUD impacting 16 million people annually. The United States (U.S.) is no exception to this epidemic [1]. Three million people in the U.S. suffer with OUD (1), with over 100,000 individuals dying from an opioid involved overdose in 2021 – a five-fold increase in opioid-related deaths compared to 1999 [2]. Indeed, since 1999, the opioid overdose epidemic has been conceptualized in three waves, with the first being overprescribing of opioid medications for pain by healthcare providers, the minimization of opioid misuse risk, and the rampant advertising of opioid drugs (e.g., OxyContin). The second wave involved the increase in the use of heroin, partially in response to lower availability of opioid pills (due to increased scrutiny over opioid prescribing) [3]. Over the past decade, synthetic opioids such has illicitly manufactured fentanyl, have predominantly driven the third wave of the overdose epidemic [4-6]. It appears we are now in the fourth wave of the overdose epidemic - characterized by a dramatic increase in overdose deaths associated with psychostimulants (laced with synthetics) [7].

In addition to the sharp rise in opioid-related deaths, the financial costs associated with the opioid epidemic cannot be overstated. The Joint Economic Committee recently estimated that opioid-related costs in the U.S. rose to nearly $1.5 trillion in 2020, representing a $487 billion increase from 2019 [8, 9]. Included in this analysis are costs related to lost productivity, health care, substance use disorder treatment, insurance, criminal justice, reduced quality of life and the value of life lost. Yet, there are unmeasured costs, including the impact of witnessing a loved one suffer from an OUD and/or to learn of their death by overdose. Further, one must consider the children of parents with an OUD. The US has seen dramatic increases in the incidence of infants born dependent on opioids due to in utero opioid exposure [10-11]. Additional costs related to OUD’s contribution to other disease onset – including HIV and hepatitis C [12] - are also difficult to quantify.

In response to this public health crisis, significant advances have been made in the effective treatment of OUD, particularly psychopharmacological interventions including methadone (synthetic opioid agonist), buprenorphine (partial opioid agonist), and naltrexone (opioid antagonist). However, there are numerous reasons why these medication treatment advances have not been sufficient to combat the current opioid epidemic. First, these medications remain underutilized in many areas due to limited access. The National Institute on Drug Abuse (NIDA) reports that less than half of substance use treatment programs in the private sector offer medications for OUD and only a third of patients in these programs who meet criteria receive medications for OUD [13]. Treatment access may also be contributing to widening health disparities, particularly the rise in opioid overdose deaths in Black communities in recent years [14]. Lack of adequate screening and identification of problematic opioid use and stigma also contribute to the gaps in access to these treatments [15-18].

Second, many critical clinical questions remain with regard to the effectiveness of medications for OUD among adolescents, particularly among adolescents who misuse fentanyl. With sublingual buprenorphine/naloxone being the only medication with FDA approval to age 16 years, youth face increased barriers in accessing these lifesaving medications. Other questions include effective initiation and dosing strategies, as well as optimal duration of treatment and strategies for discontinuation of treatment. It is also vital to better understand how to integrate medication management with psychotherapy to increase treatment retention and compliance among adolescents [19-21].

Third, treatment interventions for OUD are primarily implemented *once an individual has developed a severe disorder*. Like most health problems, shifting attention to much earlier in the etiological process can lend itself to a more cost-effective approach by prevention future morbidity and mortality. McLellan, Koob, and Volkow recently highlighted the importance of early intervention before the onset of a use disorder, conceptualizing a stage of “pre-addiction” [22]. Only addressing those with moderate-severe OUD or those who overdose fails to adequately address the increasing number of individuals in the process of developing opioid-related problems and who are at increased risk of opioid overdose. In sum, what has been sorely lacking is adequate resources toward prevention and early intervention as critical public health strategies to curtail the opioid epidemic.

To this end, every public health indicator signals the need for more robust *prevention* of OUD. Existing prevention strategies have primarily targeted the first wave of the opioid epidemic with prescription drug monitoring programs, prescriber/dispenser education, and drug take-back programs. These approaches have helped reduce availability of pharmaceutical opioids, but they do not address an individual’s risk for developing OUD, nor the sharp rise in illicitly manufactured fentanyl and the current (fourth) wave of opioid-related deaths and increases use psychostimulants (laced with synthetics) [7]. There is critical need for prevention interventions that target individual level risk factors for opioid misuse, OUD, and overdose.

Rigorous epidemiological and clinical studies have demonstrated that the presence of other non-opioid substance use disorders (SUDs; e.g., cannabis, alcohol, etc.) is the strongest, most reliable individual level predictor of the subsequent development of OUD. Specifically, data indicate rates of past-year opioid misuse are significantly higher among adolescents and young adults with either cannabis or alcohol use problems compared to same-age peers who do not have a SUD [15]. Indeed, 90% of adults with a SUD began using substances during adolescence [16]. Further, while the most recent Monitoring the Future data indicate that the national prevalence of past year substance use remains steady among adolescents, the adolescent overdose rate continues to climb [17]. Specifically, the overdose rates rose by a startling 90% among adolescents 14-18 years in the U.S. in 2021 and yet another 20% in 2022 [17]. As with adults, this sharp rise in overdose deaths is largely attributed to increasing availability of illicitly manufactured fentanyl and other synthetics, including in combination with other opioid and non-opioid drugs in pressed pills [17]. Tanz et al. (2022) recently reported that opioid use was involved in 90% of the deaths of youth 10-19 years in 2019-2021, with illicit fentanyl and other synthetics accounting for 59% of overdose deaths among this age group [18]. This has resulted in higher risk of overdose among youth with minimal opioid exposure and who may not meet criteria for OUD, further underscoring the public health significance of broadening overdose prevention efforts to adolescents engaging in risky substance use behavior.

Thus, targeted interventions for adolescents with substance use problems, including for non-opioid drugs, is critical to prevent the onset of OUD and turn the tide of the overdose epidemic. In line with this call to action to move toward earlier intervention as a public health strategy, we propose the following recommendations:

1. Universal screening for substance use should be implemented in frontline healthcare settings for children as young as 11-years-old (e.g., pediatrics, dental, emergency). Health and dental clinics, as well as mental health agencies that serve adolescents should ensure screening for substance use and SUD is part of the standard assessment-and that SUD services are offered as indicated (19). Indeed, research has shown that embedding empirically-supported screeners, such as SBIRT (Screening, Brief Intervention, Referral to Treatment), into medical settings can have a significant positive impact on service initiation (20). To meet the needs of all youth with SUD, availability of and equitable access to empirically supported treatment should be considered among the highest priorities in communities. It is also vital that resources dedicated to the opioid epidemic be made available to expand access to evidence-based treatments.

2. Substance use risk reduction interventions should be delivered as secondary prevention strategies among youth at risk of developing a SUD. These risk factors include traumatic events and other adverse childhood experiences (including youth who have lost a caregiver to an opioid overdose), other mental health comorbidities (e.g., depression, bipolar disorder, and attention deficit/hyperactivity disorder), a prior personal history of substance use, family history of SUD, low caregiver monitoring, affiliating with peers who use substance, and living in communities with easy access to substances [21-23]. For example, Risk Reduction through Family Therapy (RRFT) has been shown to be effective in significantly reducing substance use problems and posttraumatic stress disorder symptoms among adolescents who have experienced trauma [21]. Among caregivers with OUD, family inclusive treatment services should be incorporated to address trauma exposure and risk for SUD trajectory in children.

3. Universal prevention strategies are critically needed to promote healthy choices during adolescence and prevent the onset of substance use. One strategy is to equip schools with evidence-based prevention and early intervention curricula, such as Project ALERT [24], that 1) provide accurate information and target common myths regarding the safety of substance use, especially during these developmental years and 2) teach realistic refusal skills, coping skills, and other healthy living skills such as sleep hygiene. It is also important to engage caregivers in education regarding effective parenting and prevention strategies, including by way of social norm campaigns to increase caregiver knowledge about the impact of non-nicotine drug use and combat perpetuating myths regarding the limited role a caregiver can play in adolescent substance use.

4. Substantial research is needed to address adolescent substance use and mental health disorders to unveil the most effective treatment approaches. In addition to the treatment of OUD, further research is needed to better understand substance misuse, overdose risk, and disorder progression. Recent publications (e.g., that discuss the concept of *pre-addiction*) [25] indicate the field is showing increasing readiness to embrace the importance of both studying and intervening with youth as a strategy to combat OUD across the lifespan.

5. These recommended clinical and prevention services require additional and sustained resources. The current opioid epidemic is occurring during a National State of Emergency for Child and Adolescent Mental Health [26]. This is due in part to increases in substance use and mental health problems, clinics limiting services for youth presenting with more severe problems, extensive wait lists at the agencies that do serve these youth, and dramatic shortages of clinicians and supervisors to implement evidence-based interventions and risk reduction strategies. Such agencies and organizations are further impeded by low reimbursement and a high degree of case management and other non-reimbursable services required to effectively engage youth and their caregivers, often resulting in challenges providing adequate compensation and retention of trained clinicians [27]. Plain and simple: The current system is not working and requires a significant overhaul and continued funding of state and federal programs that provide vital infrastructure and resources. Oversight of the application of these funds and programs at the local, state, and federal level should occur through committees that include representation from those with lived experience and all communities to be served. Careful attention should be paid to ensure that these enhanced funds work to eliminate disparities in terms of evidence-based service access.

6. In following these recommendations above, thoughtful and continuous efforts should be dedicated to the de-stigmatization of the disease of substance use disorders-through purposeful language selections (e.g., non-blaming,person-first) and public psychoeducation with medically accurate, science-based information [28]. Reduction of stigma will increase the likelihood that youth and caregivers feel comfortable endorsing substance use during screening as applicable and seeking treatment as needed.

In conclusion, the opioid epidemic is proving to be one of the costliest and most challenging public health problems of our time. It is critical that we strategically incorporate efforts to intervene earlier in the OUD etiologic pipeline. This calls for making sizeable investments in prevention, early intervention, and treatment among adolescents who use substances. It is also vital that research is conducted in parallel to evaluate where these resources and efforts translate into the best public health yield and how prevention and intervention strategies are best implemented among youth. Effective, far-reaching change that will truly “move the needle” will also require a significant overhaul of the system, including increases in reimbursement of services to support clinician and vital ancillary staff services [29]. The field may be a bit late to the party on this youth-focused approach-but, as they say, better late than never.
